# MicroRNA-96 Promotes Vascular Repair in Oxygen-Induced Retinopathy—A Novel Uncovered Vasoprotective Function

**DOI:** 10.3389/fphar.2020.00013

**Published:** 2020-02-03

**Authors:** Michel Desjarlais, Maëlle Wirth, José Carlos Rivera, Isabelle Lahaie, Rabah Dabouz, Samy Omri, Pakiza Ruknudin, Celine Borras, Sylvain Chemtob

**Affiliations:** ^1^ Department of Ophthalmology, Maisonneuve-Rosemont Hospital Research Center, University of Montreal, Montreal, QC, Canada; ^2^ Departments of Pediatrics, Ophthalmology and Pharmacology, Centre Hospitalier Universitaire Sainte-Justine Research Center, Montreal, QC, Canada

**Keywords:** micro-RNA (miRNA), vascular degeneration, vascular repair and angiogenesis, oxygen-induced retinopathy (OIR), endothelial dysfunction

## Abstract

**Background and Aims:**

Vascular degeneration is a hallmark in the pathogenesis of oxygen-induced retinopathy (OIR). Dysregulation of microRNAs (miRNAs), key regulators of genes expressions, has been implicated in the regulation of ocular angiogenesis. However, miRNAs specific functions in impaired vascular development during OIR are poorly understood. Herein, we identified miR-96 as one of the most highly expressed miRNAs in the retina and choroid during vascular development and investigated the potential role of miR-96 on microvascular degeneration in a rat OIR model.

**Methods and Results:**

Next generation sequencing (NGS) and qRT-PCR analysis showed that miR-96 maintain high levels of expression during ocular vascular development. Nevertheless, miR-96 was significantly downregulated in the retina and choroid of OIR rats (80% O_2_ from P5 to P10) during the phase of microvascular degeneration. Similarly, human retinal microvascular endothelial cells (HRMEC) subjected to hyperoxia (80% O_2_) showed a significant downregulation of miR-96 evaluated by qPCR. Interestingly, HRMEC supplemented with miR-96 regulated positively the expression of several key angiogenic factors including VEGF and ANG-2. To explore the angiogenic activity of miR-96 on HRMEC, we performed a gain/loss of function study. In a similar way to hyperoxia exposure, we observed a robust angiogenic impairment (tubulogenesis and migration) on HRMEC transfected with an antagomiR-96. Conversely, overexpression of miR-96 stimulated the angiogenic activity of HRMEC and protected against hyperoxia-induced endothelial dysfunction. Finally, we evaluated the potential vasoprotective function of miR-96 in OIR animals. Rat pups intravitreally supplemented with miR-96 mimic (1 mg/kg) displayed a significant preservation of retinal/choroidal microvessels at P10 compared to controls. This result was consistent with the maintenance of physiologic levels of VEGF and ANG-2 in the OIR retina.

**Conclusion:**

This study demonstrates that miR-96 regulates the expression of angiogenic factors (VEGF/ANG-2) associated to the maintenance of retinal and choroidal microvasculature during physiological and pathological conditions. Intravitreal supplementation of miR-96 mimic could constitute a novel therapeutic strategy to improve vascular repair in OIR and other ischemic retinopathies.

## Introduction

Ocular vascular degeneration is an initial feature in the pathogenesis of several types of ischemic retinopathies, including retinopathy of prematurity (ROP) (Rivera et al., 2017). ROP is a well-known visual impairment in premature babies, characterized by an incomplete vascularization of the peripheral retina that causes ischemia and leading to an abnormal and excessive compensatory angiogenic response (pathological neovascularization [NV]) (Sapieha et al., 2010; Wu et al., 2013; Rivera et al., 2017a). However, inner retinal vasculature is not the only affected area during OIR. Recent evidences showed that choroidal thinning is present in adolescents and adults formerly affected with ROP (Wu et al., 2013; Erol et al., 2016; Rivera et al., 2017b). Similarly, our group also detected a sustained choroidal thinning in different animal models of ROP (Shao et al., 2011; Zhou et al., 2016; Beaudry-Richard et al., 2018). To date, several studies have focused their effort in developing novel anti-angiogenic monotherapy against the aberrant NV, by using anti-growth factors such as anti-VEGF, -FGF, -IGF and their receptors (Cabral et al., 2017). However, a small number of studies have focused their efforts on attempting to promote and improve revascularization during the initial phase of ischemia, a strategy that should potentially limit subsequent pathologic NV.

Several mechanisms involved in the control of the angiogenic signaling response and vascular repair during ROP are reported to be altered, including those related to the expression of microRNAs (miRNAs), key regulators of gene expression (O'Connell et al., 2010; Caporali and Emanueli, 2012; Paul et al., 2018). miRNAs are a family of small non-coding RNAs (20–25 nucleotides) involved in post-transcriptional regulation of genes by inhibiting protein translation or by degrading specific mRNA with a perfectly complementary target binding sequence (miRNA/mRNA). miRNAs regulate a wide range of targets essential for various biological processes such as growth, apoptosis/survival, immune response, cell migration, proliferation and stem cell function (O'Connell et al., 2010; Caporali and Emanueli, 2012; Paul et al., 2018). Physiologic expression levels of miRNAs are altered in several pathological states, suggesting their critical role in the progression of various diseases such as cancer (Hosseinahli et al., 2018), cardiovascular diseases (Zhou et al., 2018), degenerative disorders (Qiu et al., 2015), and retinopathies (Wu et al., 2012; Wang et al., 2017; Desjarlais et al., 2019b).

miRNAs play a key regulatory role to maintain functional activity of endothelial cells (ECs), in particular in the control of angiogenic capacity (Desjarlais et al., 2017; Fernández-Hernando and Suárez, 2018). Endothelial cell dysfunction is characterized by impaired capacity to proliferate, migrate and form tubules—critical components associated with insufficient post-ischemic revascularization (Isner and Asahara, 1999; Desjarlais et al., 2017). Some studies have shown changes in the expression of miRNAs in oxygen-induced retinopathy model (OIR). Our group recently reported a significant alteration in the expression level of several miRNAs in the retina and choroid associated with the vascular degeneration phase in a model of OIR in rats (Desjarlais et al., 2019b). However, the individual function of some specific miRNAs associated to the retinal and choroidal vascular degenerative process in OIR has not been investigated.

Next generation sequencing (NGS) revealed miR-96 as abundantly expressed during oculo-vascular development. Although implicated in development of the hindbrain (Schluter et al., 2018), and proliferation of cancer cells (Lin et al., 2010), its role in angiogenesis is unknown. In the present study, we identified miR-96 as an important vasoprotective miRNA that modulates VEGF and Ang-2 expression in the retina and choroid during physiological conditions. miR-96 downregulation was highly associated with impaired angiogenic process in endothelial cells exposed to hyperoxia *in vitro* and *in vivo* during vasoobliteration in OIR. Intravitreal supplementation of miR-96 prevented endothelial cell impairment induced by hyperoxia and microvascular degeneration in the retina and choroid during OIR. Altogether, these results suggest that miR-96 supplementation could be considered as a novel therapeutic strategy to improve and rescue retinal/choroidal vascular repair by promoting VEGF/Ang2 signaling in ischemic retinopathy.

## Materials and Methods

### Animal Care

All animal experimental procedures were performed with strict adherence to the ARVO Statement for the Use of Animals in Ophthalmic and Vision Research and approved by the Animal Care Committee of the Hospital Maisonneuve-Rosemont in accordance with guidelines established by the Canadian Council on Animal Care.

### 50/10 Oxygen-Induced Retinopathy (OIR) Model in Rats

Cycling oxygen-induced retinopathy (OIR) in rats was used to evaluate the expression profile of miR-96 in the retina and choroid during the pathological progress of this disease. This model is characterized by a first phase of progressive microvascular degeneration that occurs between postnatal (P) days 1 and 14 (during cycling oxygen (50–10% every 24 h), followed by a second phase of abnormal pathological NV that take place when pup rats are returned to room air between days 14 and 18 as previously described (Rivera et al., 2015; Desjarlais et al., 2019b). Briefly, a few hours after birth, litters of Sprague–Dawley albino rats (Charles River, St. Constant, QC, Canada) were placed with their mothers in an oxygen-regulated environment (OxyCycler A820CV; BioSpherix, Ltd., Redﬁeld, NY, USA) adjusted to alternate between 50 and 10% oxygen every 24 h for 14 days (OIR group). At P14, rat pups were transferred to room air (21% O_2_) for 3 days (P17). Age-matched normoxic control rat pups (NOR) were kept in room air (21% O_2_) throughout the experiment. Retinal and choroidal samples were isolated at P7, P14 and P17 from OIR and control animals and evaluated by Next Generating Sequencing and qPCR as described (Desjarlais et al., 2019b).

### Vaso-Obliteration Model (80% Constant Oxygen)

The angiogenic function of miR-96 in the retina and the potential vasoprotective effects of miR-based therapy during vascular degeneration were evaluated using a model favoring vaso-obliteration in rats (Rivera et al., 2015). Retinal vaso-obliteration (VO) was induced in Sprague–Dawley rat pups subjected to constant hyperoxia (80% O_2_) in chambers controlled by a computer-assisted Oxycycler (BioSpherix, Ltd.) from P5 to P10. Age-matched normoxic control rat pups (NOR) were kept in room air (21% O_2_) throughout the experiment. Thirty minutes before hyperoxia exposure at P5, the OIR pups were anesthetized and intravitreally injected or not, with 1 µl (1 mg/kg) of miR-96-5p mimic, or miR-mimic negative control (scrambled) (GE Healthcare Dharmacon, Lafayette, CO). This dose was chosen based on preliminary experiments showing the dose-range for optimal transfection efficiency in tissues (Desjarlais et al., 2017). miRNAs were administered in a mixture solution of Invivofectamine 3.0 (Thermo Fisher, ON, Canada) according to the manufacturer's recommendations. For molecular analysis, the OIR and control animals were euthanatized, and retinas collected at P6, P8, P10. Vessel immunostaining analysis (retinal flat mounts and cryosection) was performed at P10.

### Molecular Analysis

#### miRNA Isolation and Next Generation Sequencing Analyses

To explore the retinal/choroidal expression profile of miR-96 compare to the other miRNAs during OIR (OIR cycling model), total RNA was extracted from retinas and choroid tissues from OIR and NOR groups at P7, and P14, using the miRNeasy mini kit (Qiagen) according to the manufacturer's protocol. Quantification of total RNA was made with a nanodrop and 1 µg of total RNA was used for library preparation. Quality of total RNA was assessed with the BioAnalyzer Nano (Agilent) and all samples had a RIN above 8. Library preparation was done with the Truseq Small RNA library preparation kit (Illumina, Cat no. RS-200-0012). Eleven PCR cycles were required to amplify libraries. Libraries were quantified with a nanodrop and the quality was assessed with the BioAnalyzer High Sensitivity (Agilent). All libraries were diluted to 10 nM, normalized and pooled (n = 5) to equimolar concentration based on Miseq v2 50 cycles using 7pM of pooled library. Sequencing was performed with the Illumina Hiseq2000 using the Hiseq Reageant Kit v3 (200 cycles, paired end) and 1.7 nM of the pooled library. Around 70 million paired-end reads were generated per sample. Quantification includes the raw read count, as well as normalized expression level as RPM values (reads per million reads mapped) to account for the variability in the library size.

#### qRT-PCT Validation of mIR-96

To validate the effects of hyperoxia on miR-96 expression, total RNA where extracted from retinas of OIR and normoxia-raised (NOR) rats (OIR-vasoobliteration model) at P6, P8 and P10, and in human microvascular retinal endothelial cells (HRMEC) subjected or not to hyperoxia (80%O_2_) for 1, 3, 6, 24 and 48h, using the miRNeasy mini kit (Qiagen) according to the manufacturer's protocol. RNA was reverse transcribed using with miScript II RT kit (Catalogue # 218161, QIAGEN, Hilden, Germany) according to manufacturer's guidelines. Real-time PCR was performed using 25 ng of cDNA sample by quantitative real-time PCR using iTaq Universal SYBR Green Supermix (BioRad) with 2 μM of miR-96-5p primers designed using Primer Bank and NCBI Primer Blast software (Alpha DNA, Montreal, Canada). Relative expression (RQ = delta/delta CT) was calculated using the detection system ABI Prism 7500 (Applied Biosystems, Foster City, CA, USA) and normalized to 18S and U6 snRNA.

### Immunohistochemistry of Retinal and Choroidal Vessels

To analyze retinal vasculature, retinal flat mount dissection was performed on the enucleated eyes fixed in 4% paraformaldehyde for 1 h at room temperature and then stored in PBS until used. The retinas were incubated overnight in 1% Triton X100, 1 mM CaCl2/PBS with the tetramethylrhodamine isothiocyanate–conjugated lectin endothelial cell marker Bandeiraea simplicifolia (1:100; Sigma-Aldrich Corp., St. Louis, MO, USA). Retinas were washed in PBS and mounted on microscope slides (Bio Nuclear Diagnostics, Inc., Toronto, ON, Canada) under coverslips with mounting media (Fluoro-Gel; Electron Microscopy Sciences, Hatfield, PA, USA). Retinas were photographed under an epifluorescence microscope (Zeiss AxioObserver; Carl Zeiss Canada, Toronto, ON, Canada), and the images were merged into a single file using the MosiaX option in the AxioVision 4.6.5 software (Zeiss). Retinal microvasculature has been quantified by the percentage of total retinal vasobliteration, as well as the vascular density of the retina located in the same central and peripheral regions (Sapieha et al., 2008; Rivera et al., 2015). For choroidal vasculature, retinal cross-sections were performed. Eyes were collected, dehydrated by alcohol, and embedded in paraffin. Sagittal sections (7 µm thick) were cut by microtome (RM 2145; Leica, Wetzlar, Germany). Posterior eyecups were frozen in optimal cutting temperature medium and stained for choroidal vessels with TRITC-conjugated tetramethylrhodamine isothiocyanate-labeled lectin (Sigma-Aldrich) in the cryosections. Sections were then visualized with an epifluorescence microscope (Eclipse E800; Nikon, Tokyo, Japan). In some experiment, cryosections of the different groups were co-stained by using rabbit antibody anti-VEGF (1:200, sc-152; Santa Cruz Biotechnology, Santa Cruz, CA, USA) by incubation overnight at 4°C in the blocking solution. Secondary antibodies such as Alexa Fluor 488 anti-rabbit (Life technologies) were used at a dilution of 1:1,000. Cell nuclei was identified with DAPI labeling. Incubation using rabbit or goat IgG as a primary antibody was conducted as a negative control. The image was split into the three-color channels (RGB Merge/split function) to obtain one image per channel.

### *Ex Vivo* Choroidal Angiogenic Sprouting Assay

Angiogenic sprouting capacity of the choroid isolated from the different groups of rats were assessed as previously descripted (Shao et al., 2013). Briefly, choroid was isolated from rat pups at P10, sectioned into 1-mm rings, and placed into growth- factor–reduced Matrigel (Fisher Scientific, New Hampshire, USA) in 24-well plates and cultured at at 37°C, 5% CO_2_ and 95% air, for 5 days in endothelial growth medium; medium 200 (Life technologies) supplemented with 10% fetal bovine serum (FBS, Wisent, St-Jean-Baptiste, QC, Canada), 100 IU/ml penicillin/0.1 mg/ml streptomycin (Wisent) and low serum growth supplement (LSGS; 2% FBS, 3 ng/ml bFGF, 10 mg/ml heparin, 1 mg/ml hydrocortisone, and 10 ng/ml EGF; Life Technologies). Photomicrographs of individual explants were taken at day 5 using an inverted phase-contrast microscopy (AxioObserver; Zeiss), and microvascular sprouting area was quantified using Image J.

### *In Vitro* Angiogenesis Analyses

#### Cell Culture

Human Retinal Microvascular Endothelial Cells (HRMECs) were purchased from Applied Biological Materials (cat #T4169) and cultured in medium 200 (Life technologies) supplemented with 10% fetal bovine serum (FBS, Wisent, St-Jean-Baptiste, QC, Canada), 100 IU/ml penicillin/0.1 mg/ml streptomycin (Wisent) and low serum growth supplement (LSGS; 2% FBS, 3 ng/ml bFGF, 10 mg/ml heparin, 1 mg/ml hydrocortisone, and 10 ng/ml EGF; Life Technologies). In some experiments, HRMECs were subjected or not to hyperoxia (80%) using oxygen monitoring chambers for different durations (1, 3, 6, 24 and 48h). HRMECs were grown at 37°C, 5% CO_2_ and 95% air, and the medium was changed every 2 days and passaged when they reached 90% confluence; passages 3–6 were only used for experiments.

#### miRNA Transfection in HRMEC

Transfections were carried out at a concentration of 50 nM using Lipofectamine RNAiMAX Reagent (Thermo Fisher, ON, Canada) according to the manufacturer's protocol and as previously described. Briefly, HRMECs were transfected 24 h after being plated in 6-well plates with the following miRs purchased from Dharmacon (GE Healthcare Dharmacon, Lafayette, CO): miRIDIAN miR mimic negative control #1, miRIDIAN miR mimic hsa-miR-96-5p, miRIDIAN antago-miR negative control #1, miRIDIAN antago-miR-96-5p. After 24 h, the transfection medium was replaced with antibiotic-free complete M200 medium and cells were subjected or not to hyperoxia (80%) for 24 h. Transfection efficiency was measured using mimic transfection control Dy547 (Dharmacon) and found to be 80–90% and also confirmed by qRT-PCR.

#### HMREC Capillary-Like Tubulogenesis on Matrigel

The angiogenic activity of HRMECs was determined using a Matrigel tube formation assay. Briefly, after transfection and exposure conditions, HRMECs were plated at a density of 30,000 cells/well in 96-well plates precoated with 50 μl of growth factor reduced Matrigel Matrix (Fisher Scientific, New Hampshire, USA) and cultured at 37°C for 6 h in complete endothelial growth medium. HRMECs were pre-transfected or not with miR-96-5p mimic, antagomiR-96-5p or appropriate miR controls for 24 h, and subjected or not to hyperoxia (80% O_2_) for 6 h after plated in Matrigel. Capillary-like tubes were observed under a light microscope. Images were obtained at 10× magnification, and all tubes and branching point were counted.

#### Migration Scratch Assay

Measurement of cell migration was performed using an adapted scratch assay in confluent HRMECs. The cells were transfected and grown to near confluence in 24-well plates and subjected or not to hyperoxia (80% O_2_). Mechanical disruption of the monolayer was realized by scraping with a pipette tip. Migration was assessed 24 h after mechanical disruption using an inverted microscope at a magnification of 200× and the area of migration and the cells density where quantified as previously descripted (Desjarlais et al., 2017; Desjarlais et al., 2019a). Six fields per well were evaluated and all experiments were performed in duplicate.

#### qRT-PCT Analyses of Angiogenic Factors Level

To explore the effects of miR-96-5p overexpression/inhibition on angiogenic factors mRNAs level in HMREC and in the rat retinas, total RNA was extracted using RNeasy mini kit (Qiagen) according to the manufacturer's protocol and was reverse transcribed using iScript-II RT kit (Qiagen) according to manufacturer's guidelines to generate cDNA. Quantitative real-time PCR reaction was performed using 25 ng of cDNA sample, 2 μM of specific primers for the selected mRNAs (Alpha DNA, Montreal, Canada) and Universal SYBR Green Supermix (BioRad). Relative expression (RQ = 2^−^^Δ^ ^Δ^ ^CT^) was calculated using the instrument detection system; ABI Prism 7500 (Applied Biosystems, Foster City, CA, USA) and normalized to b-Actin and GAPDH.

#### Western Blot Analysis

Protein levels of VEGF, Ang2 and FGF-2 were analysed by Western blots in the retina of the different groups of rats and in HMREC extracts. For total protein extraction, isolated retina where rinsed in PBS, snap-frozen in liquid nitrogen, and stored at −80°C until use. Whole-cell protein extracts were obtained after homogenization of the retina of the different groups of rats in ice cold RIPA buffer (pH = 8) containing 50 mM Tris–HCL, 150 mM NaCl, 5 mM EDTA, 1% Triton 100×, 0.5% sodium deoxycholate, 0.1% SDS with a cocktail of proteases and phosphatases inhibitors (MiniComplete, PhosphoStop and PMSF, Roche, Bâle, Switzerland). HRMECs were lysed with 50 µl of RIPA lysis buffer per well in 6-well plates, harvested and sonicated. 50 µg of protein per retina homogenate sample and 20 µg of protein per cell lysate sample were separated on an SDS-polyacrylamide gel and electroblotted on nitrocellulose membranes. Non-specific binding sites were blocked with 5% BSA for 1 h. The membranes were probed overnight at 4°C with the following antibodies: rabbit antibody VEGF (1:500, sc-152; Santa Cruz Biotechnology, Santa Cruz, CA, USA), goat antibody Ang2 (1:1,000, ab10601; ABCAM), mouse antibody FGF-2 (1:1000, ab181; ABCAM) or GAPDH (1:2,000, ab181602; ABCAM). Membranes were then washed three times for 10 min with TBS-T and incubated with secondary antibodies for 1 h with 1:2,500 horseradish peroxidase (HRP)-conjugated anti-mouse or 1:2,000 HRP anti-goat or anti-rabbit secondary antibodies (Millipore). Specific proteins were detected by chemiluminescent reaction (GE Healthcare, Piscataway, NJ) by membranes exposure with LAS-3000 imager. Protein expression was quantified using ImageJ and the results are expressed as density values normalized to the loading control (GAPDH).

### Statistical Analysis

All results are presented as mean ± SEM. Statistical significance was evaluated by a one- or two-way ANOVA followed by a Bonferroni *post hoc* test. A value of *P < *0.05 was interpreted to denote statistical significance.

## Results

### miR-96 Is Highly Expressed During Vascular Development But Downregulated During Hyperoxia-Induced Vessel Degeneration

**Figure 1:**
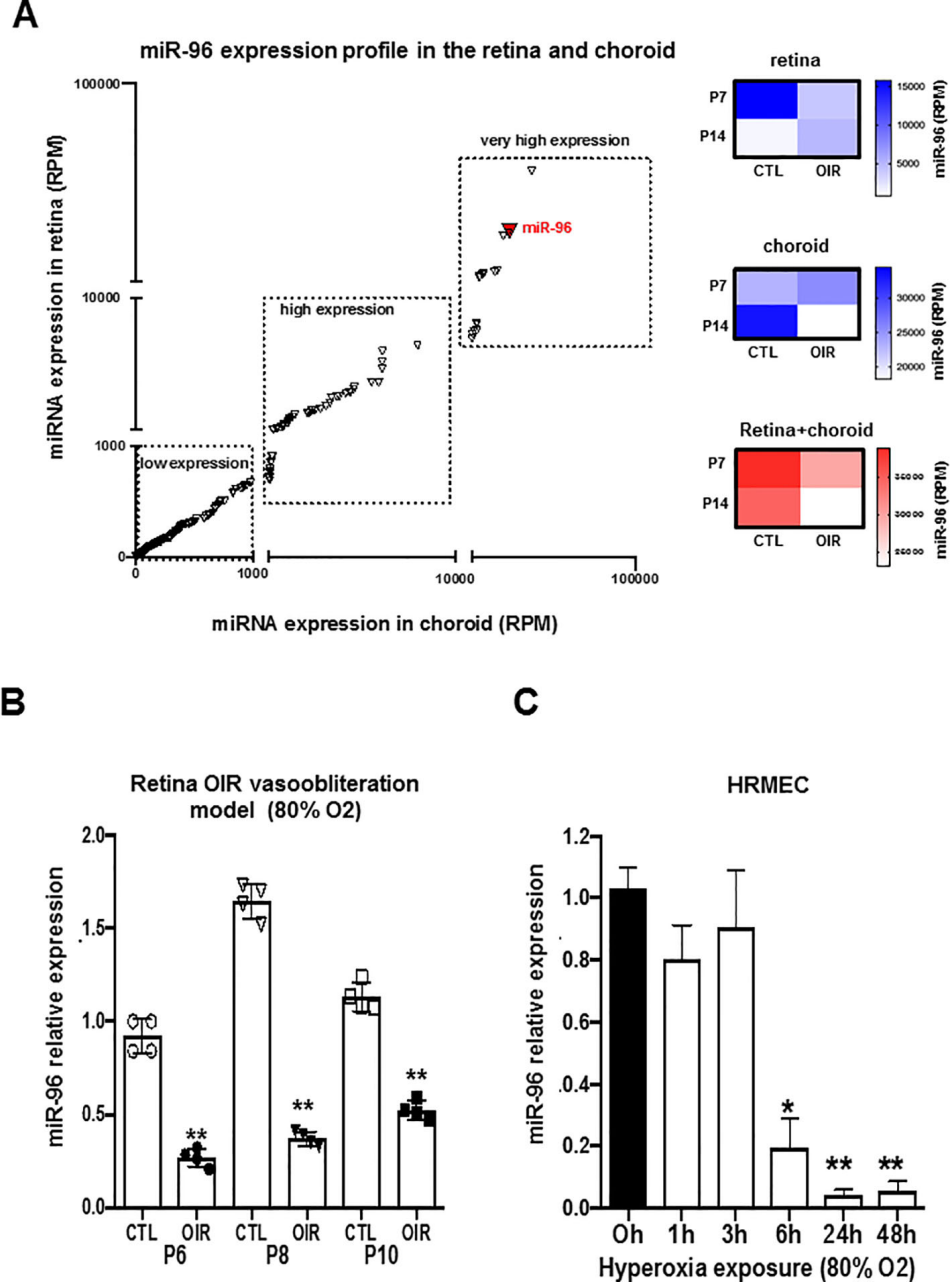
miR-96 expression is downregulated in the retina and choroid of OIR rat and in human retinal endothelial cells subjected to hyperoxia. **(A)** NGS analyses showing miR-96 expression profile (RPM) in the retina and choroid during the vascular development of normoxic control rats vs OIR rats (OIR cycling-O_2_ model). **(B, C)** qRT-PCR validation analysis of miR-96 expression in the retina of normoxic control rats vs OIR rats (vasoobliteration: constant-O_2_ model) and respectively in Human Retinal Microvascular Endothelial Cell (HRMEC) subjected to hyperoxia (80% O_2_). Data are mean ± SEM. *P < 0.05 or **P < 0.01 vs CTL (control). N = 4–5/group.

We evaluated the expression profile of miR-96 in retinal and choroidal samples of animals exposed to normoxic (NOR) and during vasoobliteration phase in 50/10 oxygen-induced retinopathy model (50/10 OIR) at P7 and P14 by using next generation sequencing (NGS) analysis. During ocular vascular development, we found miR-96 to be highly expressed in the retina at P7 and choroid at P14 in normoxic animals (**Figure 1A**). However in 50/10 OIR animals, miR-96 was profoundly downregulated in the retinal/choroidal tissues at all time points evaluated (**Figure 1A**). To better validate the effect of hyperoxia on miR-96 expression, we also analyzed its levels of expression in the rat retina using a different vasoobliteration OIR model, specifically by constant exposure to 80% O_2_. qRT-PCR analyses revealed that miR-96 expression was also significantly reduced in 80% OIR retina at P6, P8 and P10, compared to control (NOR) rats (**Figure 1B**). To assess whether miR-96 expression specifically affects endothelial cells during hyperoxia, human retinal microvascular endothelial cells (HRMEC) were subjected to 80% oxygen for different periods of time (1, 3, 6, 24 and 48 h). Hyperoxia markedly suppressed miR-96 expression (**Figure 1C**). These results infer that decreased miR-96 levels may be associated with impaired endothelial cell function during OIR.

### miR-96 Positively Regulates the Expression of Several Angiogenic Factors, Including VEGF, FGF-2 and Ang2, in Endothelial Cells

**Figure 2:**
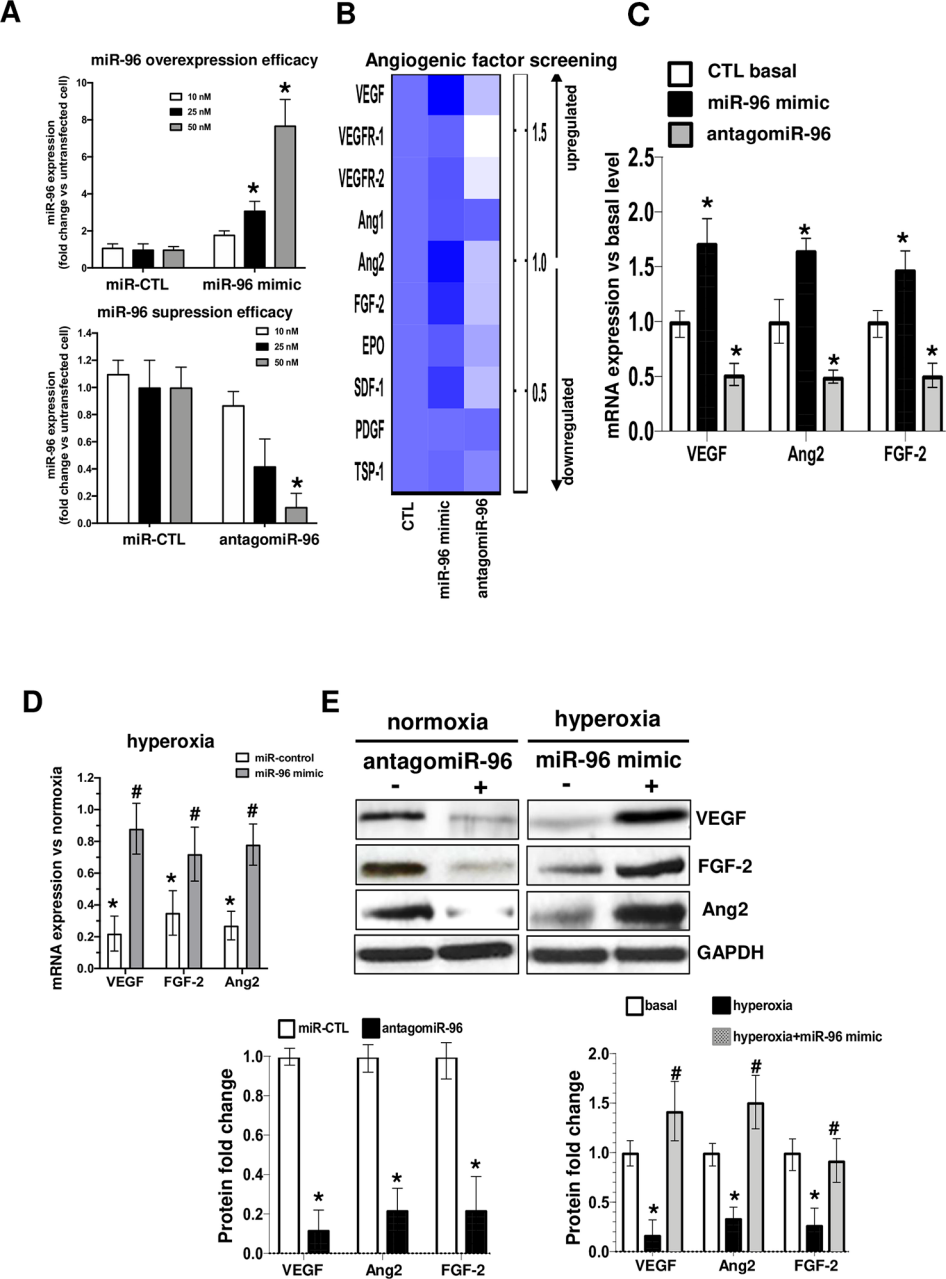
miR-96 regulate simultaneous the expression level of VEGF/FGF-2/Ang2 in human retinal endothelial cells. To explore the regulatory function of miR-96 on HRMEC angiogenic factor production, cells were pre-transfected or not with 50 nM of miR-control (scrambled), miR-96 mimic or antagomiR-96 for 24 h, and subjected or not to hyperoxia (80% O2) for an additional 24 h. **(A)** qRT-PCR analyses of miR-96 transfection efficacy, and **(B, C)** screening of angiogenic factor expression modulated by miR-96. **(D, E)** qRT-PCR analyses of miR-96 protective effects in HMREC subjected to hyperoxia **(D)** and western blot validation and compiled histogram analysis of the regulatory role of miR-96 on VEGF, FGF-2 and Ang2 proteins expression levels. N = 3–4 experiments. Data are mean ± SEM. *P < 0.05 vs CTL (control) or ^#^ *P <* 0.05 vs hyperoxia.

Based on expression profile of miR-96 in OIR we proceeded to determine if relevant angiogenic factors were regulated by miR-96, and whether maintaining miR-96 expression can prevent endothelial cell survival during OIR. Recent studies have reported an important correlation between miR-96 levels and tumor angiogenesis (Hong et al., 2016; Shi et al., 2017; Iwai et al., 2018). However, its potential role in OIR remains unknown. We investigated the effects of miR-96 up or down regulation on the expression of different angiogenic factors. We first determined the expression efficacy of miR-96 mimic or antagomiR-96 at different concentrations (10, 25 and 50 nM) for 24h in transfected HRMECs (**Figure 2A**). Transfection was highly efficient on endothelium at 50 nM for both miR-96 mimic (overexpression) and antagomir-96 (suppression), and therefore, we used this concentration in all our experiments. We next evaluated if miR-96 mimic (50 nM) in cultured HRMECs affects the expression of angiogenic growth factors. Interestingly, increased miR-96 induced simultaneously and significantly the mRNA expression of several pro-angiogenic factors including, VEGF, Ang2, FGF-2, SFD-1 and VEGFR2 (**Figure 2B, C**). Conversely, inhibition of miR-96 expression by using an antagomiR (50 nM) downregulated expression of these factors (**Figure 2B, C**). In addition, miR-96 mimic reversed hyperoxia-induced mRNAs (**Figure 2D**) and protein suppression of VEGF, Ang2 and FGF-2 (**Figure 2E**). Hence, miR-96 is an important player in the control of gene expression of growth factors essential for maintenance and signaling on endothelium.

### miR-96 Regulates the Angiogenic Function of HRMEC and Protects Against Hyperoxia-Induced Endothelial Dysfunction

**Figure 3:**
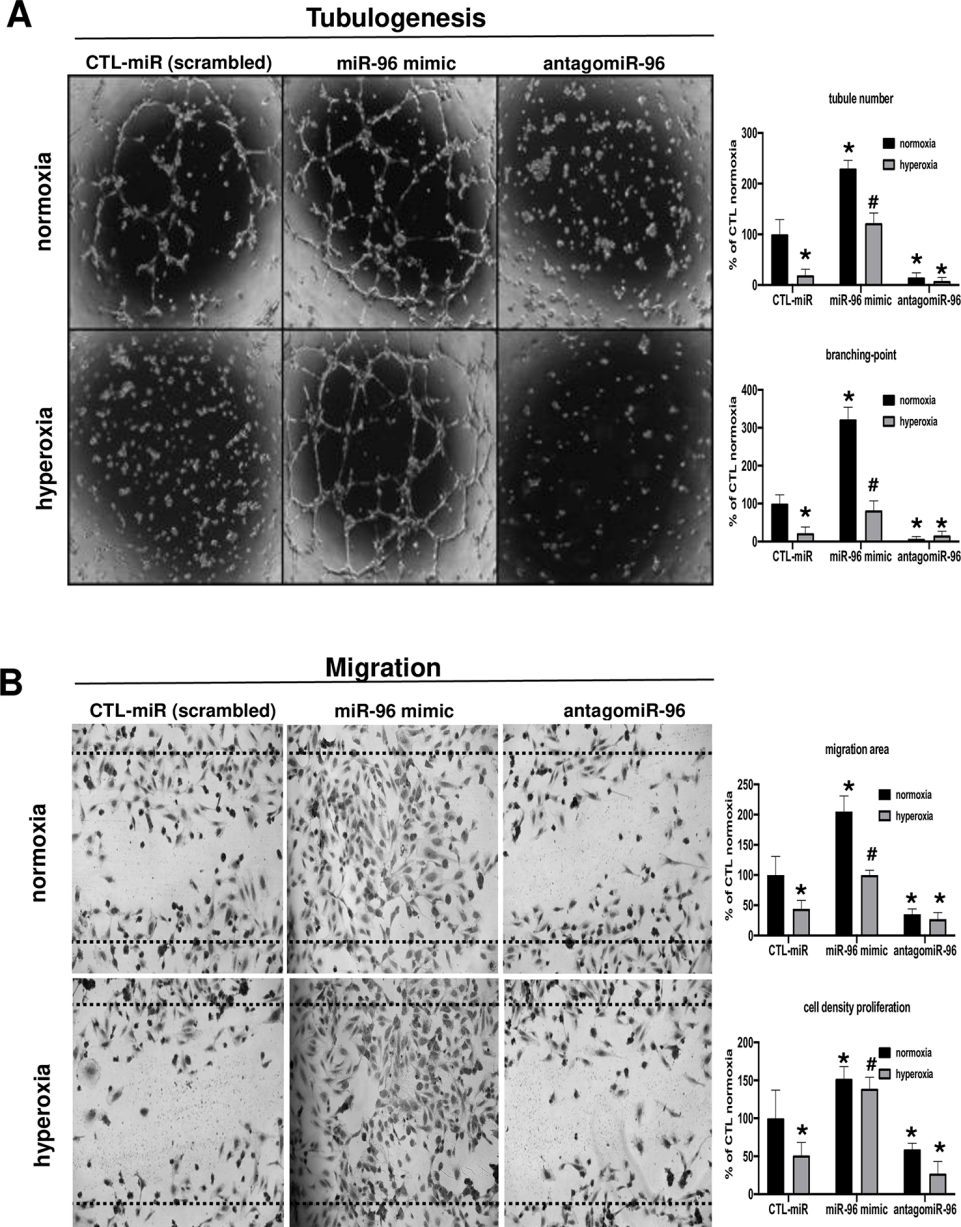
Suppression of miR-96 leads to endothelial dysfunction, and miR-96 supplementation protects angiogenic properties of endothelial cells against hyperoxia. **(A)** *In vitro* evaluation of angiogenesis (tubulogenesis) using HRMEC cultured in Matrigel, and **(B)** cell migration scratch assay of HRMEC treated or not with a miR-control (scrambled), miR-96 mimic or antagomiR-96, and exposed or not to hyperoxia (80% O_2_). Data are mean ± SEM. *P < 0.05 vs CTL (control) or ^#^ *P <* 0.05 vs hyperoxia. N = 3–4 experiments.

The next step was to investigate the potential protective effect of increased miR-96 in tube formation and migration assays *in vitro* during hyperoxic exposure. We performed a gain and loss-of function experiment by transfecting HRMECs with a miR-96 mimic or antagomir-96 in normoxic or hyperoxic conditions. In normoxic conditions, we found that augmented miR-96 significantly increased tubulogenesis (**Figure 3A**, upper panel) and endothelial cell migration (**Figure 3B**, upper panel). Conversely, miR-96 antagomir resulted in failed HRMEC tubulogenesis and migration (**Figures 3A, B**, bottom panels), consistent with that seen during hyperoxia when miR-96 was down-regulated (**Figures 1C** and **2D**); this inhibitory effect of antagomir-96 is likely contributed at least in part by suppression of VEGF/Ang2/FGF-2 proteins during hyperoxia (**Figure 2D**). On the other hand, treatment with miR-96 mimic significantly maintained endothelial cell tube formation and migration even under hyperoxic conditions (**Figures 3A, B**, bottom panel), probably also due to preservation of VEGF/Ang2/FGF-2 (**Figure 2D**).

### Intravitreal Administration of miR-96 Prevents Retinal and Choroidal Vasoobliteration in Oxygen-Induced Retinopathy Rats

**Figure 4:**
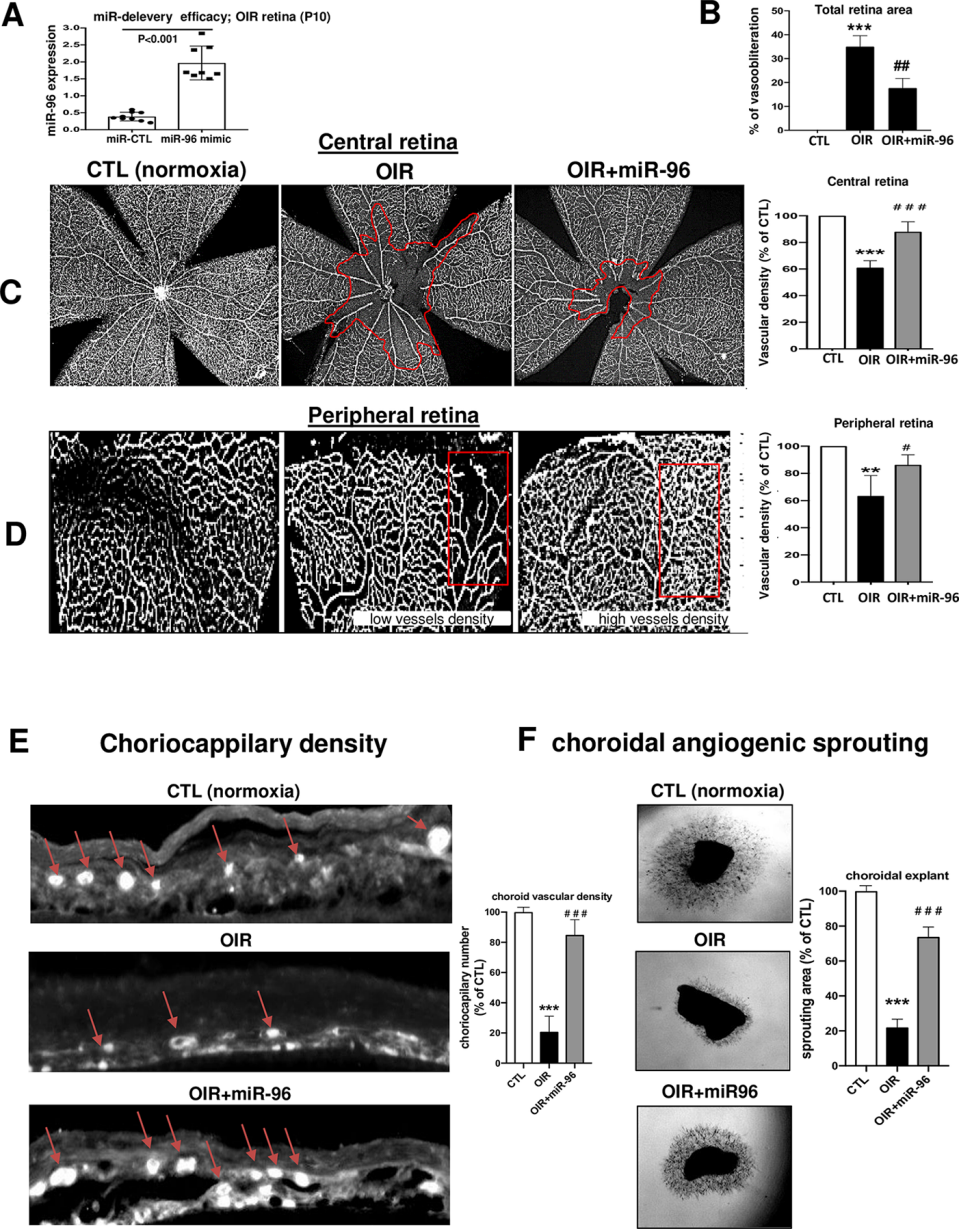
Intraocular miR-96 (mimic) prevents retinal and choroidal vascular decay and facilitates revascularization in OIR model. OIR-subjected rats were injected intravitreally with a single dose (1 mg/kg) of miR-control or miR-96 mimic at P5 before hyperoxia exposure. Animals were then subjected to constant hyperoxia (80% O_2_) until P10. **(A)** qRT-PCR analysis showing miR-96 treatment efficacy in raising miR-96 expression. **(B)** Quantification analyze of total retinal vasoobliteration in OIR-subjected rats treated or not with miR-96 mimic. **(C, D)** Representative flatmount images of central and peripheral vascularization; histogram on right show compiled analysis. **(E)** Isolectin staining of sub-retina showing choroidal vessels. **(F)** Photographic image of choroid explant vascular sprouting in Matrigel after 5 days of incubation, in tissues from normoxia- and OIR-raised animals treated or not with miR-96. Data are mean ± SEM. **P < 0.01 or ***P < 0.001 vs CTL; ^#^P < 0.05 or ^##^P < 0.01 or ^###^P < 0.001 vs OIR. N = 6–8/group.

**Figure 5:**
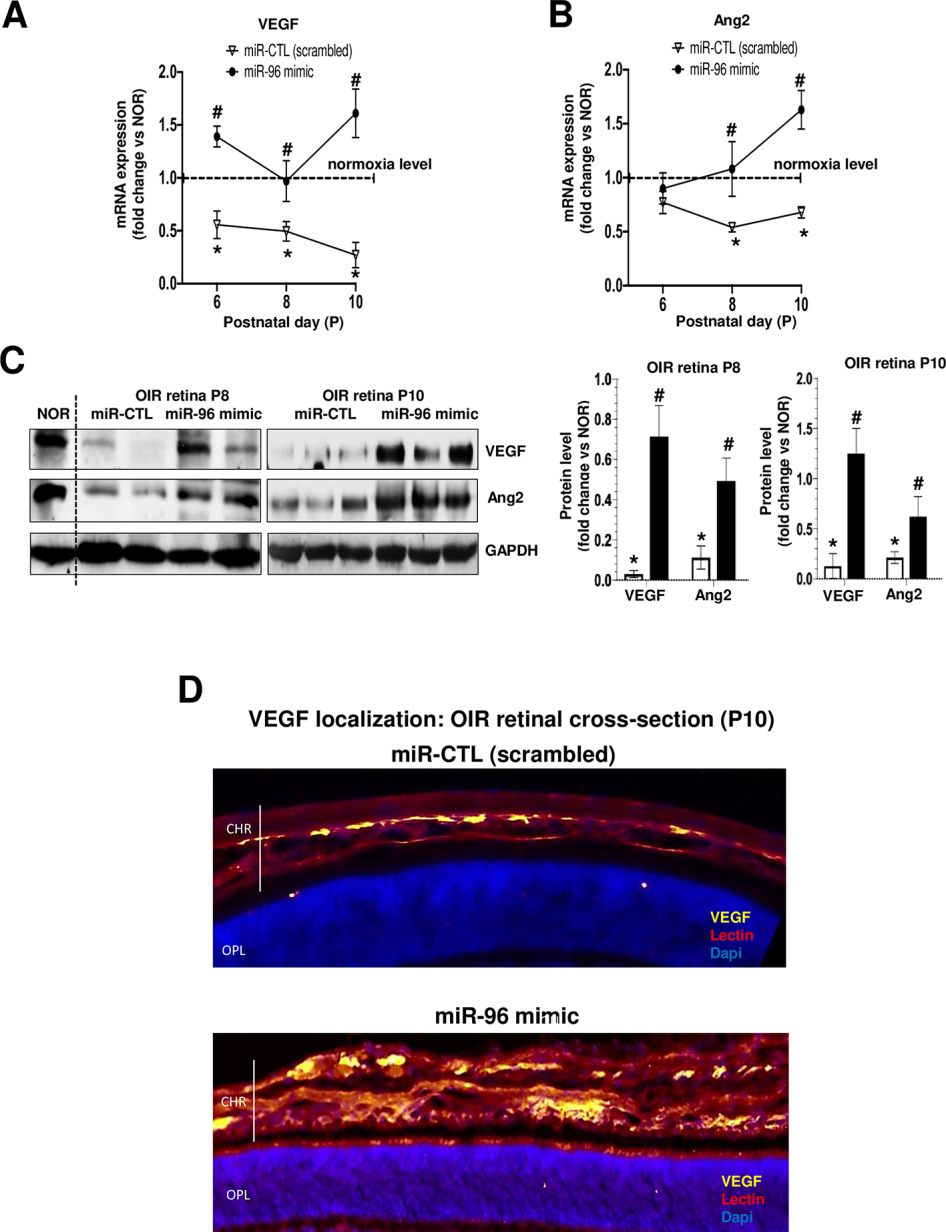
miR-96 supplementation restores VEGF and Ang2 levels in retina of OIR-subjected rats. OIR rats where intravitreally injected with a single dose (1 mg/kg) of miR-CTL or miR-96 mimic at P5 before hyperoxia exposure. After treatments, the animals were subjected to constant hyperoxia (80% O_2_) until P10. **(A, B, C)** Respectively, qRT-PCR and western blot analysis of VEGF and Ang2 expression in the retina of OIR-subjected rats treated or not with miR-96 mimic. **(D)** Immunostaining of VEGF in retinal cross-section reveals its expression co-localized on endothelium (lectin-staining) in OIR-subjected rats treated or not with miR-96 mimic. *P <0.05 vs CTL or ^#^vs OIR. N = 6–8 retinas/group.

We evaluated if miR-96 mimic can preserve retinal and choroidal vessel integrity during OIR. miR-96 was maintained increased (till at least P10) following its intravitreal administration at P5 (**Figure 4A**). Retinal and choroidal microvascular decay is a feature of the first phase of OIR (Rivera et al., 2015; Zhou et al., 2016; Desjarlais et al., 2019b), as appreciated by an increase of total retinal vasoobliteration (**Figure 4B**), central avascular area (**Figure 4C**) and reduced microvascular density in the peripheral retina (**Figure 4D**) after 5 days of exposure to hyperoxia (from P5 to P10); this was also associated with reduced choroidal thickness (**Figure 4E**). Interestingly, choroids from animals subjected to OIR exhibited a limited angiogenic ability (**Figure 4F**). Supplementation of miR-96 mimic (1 mg/kg, administered intravitreally) 30 min before hyperoxic exposure, significantly prevented retinal and choroidal vasoobliteration (**Figures 4B–E**). Moreover, miR-96 mimic allowed the choroid of OIR-subjected rats to retain an angiogenic property (as seen in Matrigel) similar to that seen in normoxia-raised rats (**Figure 4F**). Consistent with physiologic angiogenic effects of miR-96, latter augmented mRNA and protein expression levels of VEGF and Ang2 in the retina of OIR rats at P8 and P10 (**Figures 5A, B, C**). Increased VEGF was also detected *in situ* in choroid of miR-96 mimic-treated animals (**Figure 5D**). Together these observations highlight a beneficial angiogenic role of miR-96 and potential therapeutic value of this miR in preventing microvascular degeneration while preserving angiogenic capacity during OIR.

## Discussion

**Figure 6:**
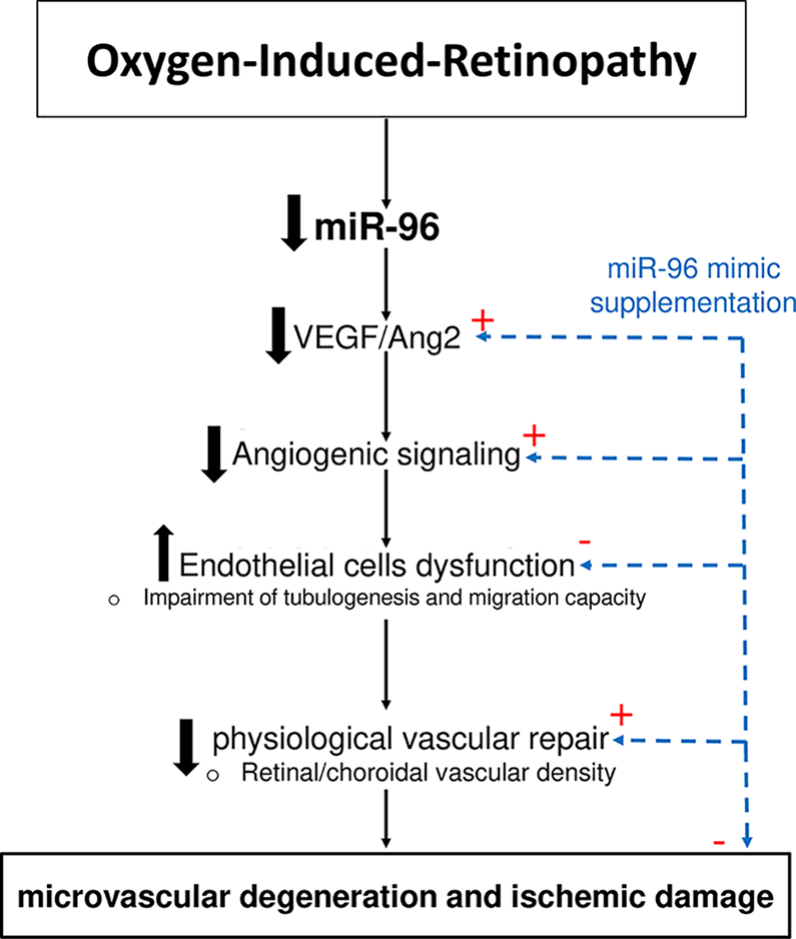
Schematic representation of vasoprotective function of miR-96 in OIR.

Although miRNAs are recognized as essential regulators of numerous genes resulting in the control of a variety of physiologic and pathologic processes (O'Connell et al., 2010; Caporali and Emanueli, 2012; Paul et al., 2018), their specific individual functions during OIR remain mostly unexplored. Recently, our group published a complete profiling of miRNAs in the retina and choroid during OIR that identified a serial of differentially expressed miRNAs during the two characteristic pathological phases in OIR (Rivera et al., 2015; Zhou et al., 2016; Desjarlais et al., 2019b). Here, we show that among these miRNAs, miR-96 was one of the most abundantly expressed miRNAs in the retina and choroid during ocular development. Furthermore, we found that miR-96 was downregulated in the retinal/choroidal tissues during the phase of vascular degeneration in OIR, inferring a potential critical role in the maintenance of ocular vasculature. Although previous studies have reported that miR-96 is essential for the normal development of the auditory hindbrain (Schluter et al., 2018), integrity and maturation of photoreceptors (Xiang et al., 2017) and proliferation of cancer cells (Lin et al., 2010), its implications in angiogenesis were still unknown. In the current study, we show that miR-96 is important for endothelial cell maintenance and proliferation. During hyperoxia-associated retinal and choroidal vascular decay, miR-96 is suppressed and along with it key angiogenic factors VEGF, Ang2 and FGF-2, resulting in curtailed endothelial function; supplementation with miR-96 (using a mimic) restored retinal and choroidal vasculature *in vivo* in model of OIR, as well as its angiogenic capacity (*ex vivo*), consistent with recently described restitution of choroidal angiogenic property by growth factors in OIR (Zhou et al., 2019). These properties of miR-96 in OIR are depicted in a schematic diagram (**Figure 6**).

miR-96 has been found to be modulated during various clinical conditions. For instance, miR-96 is upregulated in the retina of streptozotocin-induced diabetes rats (Wu et al., 2012) and in the plasma of type-2 diabetic patients (Yang et al., 2017), while miR-96 is downregulated in the RHO-mouse retina, a model for common form of inherited blindness (Palfi et al., 2016). In addition, hypoxic conditions in cancer cells have shown to modify the expression profiling of miRNAs (Shen et al., 2013; Bandara et al., 2017). In breast and prostatic cancer cell lines, miR-96 has been found to be increased by hypoxia and play a role in the regulation of autophagy process (Ma et al., 2014; Shi et al., 2017). In another study a miR-183-96-182 cluster overexpressed the master regulator of angiogenesis HIF-1a augmenting tumour progression and angiogenesis (Li et al., 2015). It is thus possible that augmented levels of VEGF/FGF-2/Ang2 in endothelial cells supplemented with miR-96 can be associated to the activation of miR-96/HIF-1 signalling; this has yet to be investigated.

Several miRNAs are modulated by reactive oxygen species (ROS) (He and Jiang, 2016; Banerjee et al., 2017), as reported in inflammation (Sonkoly and Pivarcsi, 2009), cardiovascular conditions including stroke, and in post-occlusion revascularization (Magenta et al., 2013; Jaksik et al., 2014; Gong et al., 2018). Hyperoxia in the newborn leads to a systematic increase in ROS (Kwak et al., 2006). We postulate that these ROS can downregulate miR-96 as occurs with other miRNAs under oxidative stress conditions (Magenta et al., 2013; Jaksik et al., 2014). For instance, miR-96 in human retinal pigment epithelial cell stimulated with hydrogen peroxide (ROS inducer), was found to be strongly downregulated (Ayaz and Dinc, 2018). Conversely, overexpression of the miR-183/96/182 cluster resulted in a decreased production of nitrite and ROS by macrophage (Muraleedharan et al., 2019) and a neuroprotective role in the brain, by positively regulating glutathione (GSH) levels, a major tissue antioxidant (Kinoshita et al., 2014). Collectively, these studies support the possibility that miR-96 can be decreased in hyperoxic/oxidative stress conditions; this does not exclude other mechanisms such as extracellular vesicles (Liu and Lu, 2015; Battaglia et al., 2019).

The present study is the first to show that miRNA-based therapy is beneficial in preserving vasculature during the (first) obliterative phase of OIR. Others have shown efficacy of miRNAs-based therapies to inhibit pathological neovascularization (Bai, Bai et al., 2011; Chen et al., 2017; Liu et al., 2019), but not in prevention of vascular decay. In this context miR-96-regulated VEGF and Ang2 play a critical role. Interestingly, studies have shown that VEGF and Ang-2 play a synergistic role in angiogenic stimulation (Bhaskar et al., 2013) and that conversely the dual inhibition of these factors leads to significant vascular failure (Coutelle et al., 2015; Regula et al., 2016). Consistently with our results, some studies have previously described a proangiogenic role of miR-96 in different cancer cell lines by targeting different genes with antiangiogenic functions. For example, miR-96 was reported to suppress PTPN9 (Hong et al., 2016), an important anti-angiogenic phosphatase involved in the inactivation of STAT3 (Yuan et al., 2010), a key proangiogenic transcriptional factor (Chen and Han, 2008). As is known a constitutive activation of STAT3 leads to upregulation of VEGF (Niu et al., 2002). Here, we observed a consistent increase on VEGF levels with miR-96 mimic stimulation. In addition, another study demonstrates that suppression of PTPN9 by miR-126 in endothelial cells in a mice model of angiogenesis and neurogenesis ischemia, induced the activation of AKT and ERK signaling, two major signaling pathway promoting cellular migration, proliferation and survival (Qu et al., 2019). As it is well known that HIF1a acting as a key pro-angiogenic transcriptional factor activated by hypoxia and translocated to the nucleus to induce the production of several endothelial growth factors, a recent study reported that miR-182, a member of miR-96/182/183 cluster, directly suppresses the expression of prolyl hydroxylase domain enzymes (PHD) and the inhibiting factor HIF-1 (FIH1) in prostatic tumor cells causing a marked increase in HIF1a activity (Li et al., 2015). Based on this finding, it's possible to speculate that miR-96 can also target varied negative regulators of HIF1a activities. Other potential antiangiogenic factors regulated by miR-96 include OXO1, FOXO3, RECK, EphrinA5 and SAMD9 (Hong et al., 2016). In this context, FOX family can be negatively regulated in part by FGF-2/IGF-2, and other FOX members (Essaghir et al., 2009), suggesting a complex autoregulation mechanism between miR-96/FOX/angiogenic factor. While RECK represses the expression of MMP-2 and MMP-9 (Chang et al., 2008), and EphrinA5 (Wang et al., 2016) is important in regulating endothelial migration, survival and proliferation (Hara et al., 2010). The precise mechanism by which miR-96 regulates VEGF/Ang2/FGF-2 needs further investigations.

In summary, the current study demonstrates for the first time that miR-96 regulates the expression of angiogenic factors (VEGF/Ang-2) associated with the maintenance of retinal and choroidal microvasculature during physiological and pathological conditions that lead to vascular decay. Supplementation of miR-96 mimic could constitute a novel therapeutic strategy to improve vascular repair in OIR and other ischemic retinopathies.

## Data Availability Statement

The raw data generated for this article can be found in NCBI using the accession number GSE129995.

## Ethics Statement

The animal study was reviewed and approved by Animal Care Committee of the Hospital Maisonneuve-Rosemont in accordance with guidelines established by the Canadian Council on Animal Care.

## Author Contributions

Conceived and designed the study: MD and SC. Wrote the manuscript: MD, SC, JR. Directed and planned the experiments: MD. Performed the experiments: MD and MW, assisted by JR, RD, IL, PR, SO, CB. Analysed the data: MD, JR, SC.

## Funding

MD is a recipient of a post-doctoral fellowship award from Hopital Maisonneuve-Rosemont and from the Fonds de Recherche en Ophtalmologie de l'Universite de Montreal. MW is a recipient of a bursary from Universite de Lorraine, Nancy, France. JR was supported by the Heart and Stroke Foundation of Canada and the Canadian Stroke Network. RD is recipient of an award of Excellence from the Vision Health Network of Quebec. SC holds a Canada Research Chair (Vision Science) and the Leopoldine Wolfe Chair in translational research in age-related macular degeneration. The study was financed by grants from Canadian Institutes of Health Research (grant number—MOP12532), March of Dimes Birth Defects Foundation, Fonds de la Recherche du Québec—Santé (FRQS)/Québec Vision Health Network (grantee: SC).

## Conflict of Interest

The authors declare that the research was conducted in the absence of any commercial or financial relationships that could be construed as a potential conflict of interest.
